# Changes in the Use of Non-nutritive Sweeteners in the Chilean Food and Beverage Supply After the Implementation of the Food Labeling and Advertising Law

**DOI:** 10.3389/fnut.2021.773450

**Published:** 2021-11-08

**Authors:** Camila Zancheta Ricardo, Camila Corvalán, Lindsey Smith Taillie, Vilma Quitral, Marcela Reyes

**Affiliations:** ^1^School of Public Health, University of Chile, Santiago, Chile; ^2^Institute of Nutrition and Food Technology, University of Chile, Santiago, Chile; ^3^Gillings School of Global Public Health, University of North Carolina, Chapel Hill, NC, United States; ^4^Escuela de Nutrición y Dietética, Facultad de Salud, Universidad Santo Tomás, Santiago, Chile

**Keywords:** Chile, food labeling, sugars, non-nutritive sweeteners, food reformulation

## Abstract

Reductions on the sugars content of the food supply have been described after the initial implementation Chilean Labeling Law, but it is unclear if sugars were replaced by non-caloric sweeteners (NNS). We evaluated changes in the NNSs use in foods and beverages after the initial implementation of the labeling law. We used longitudinal data on packaged foods and beverages collected in six major supermarkets and three candy distributors in Santiago, Chile, in January–February 2015/2016 and in January–February 2017. We included in the analysis beverages, dairy-based beverages, yogurts, breakfast cereals, desserts and ice creams, candies and sweet confectioneries, and sweet spreads with a market share ≥1% of their food groups (*n* = 999). We compared the use of any NNS, the number of different NNSs used, and the combined use of NNSs and ingredients adding sugars using non-parametric tests for matched samples. We evaluated the association between a reduction in sugars and starting NNS use in the post-implementation period using negative binomial regression. The use of any NNS increased from 37.9 to 43.6% (*p* < 0.001) after the law's implementation, NNSs increased among beverages, dairy-based beverages, yogurts, and desserts and ice creams (*p* < 0.05), driven mostly by increases in sucralose and stevia use (*p* < 0.05). We found that reformulated products that reduced the amount of sugars below the law's cutoff were more likely to start using an NNS in the post-implementation period (prevalence ratio: 12.1; 95%CI: 7.2–20.2; *p* < 0.001). Our results suggest that NNSs likely replaced sugars after the initial implementation of the law. Further analyses should explore how these changes may impact NNS consumption.

## Introduction

In Chile, as in other parts of the world, the prevalence of obesity is increasing. In 2016, 33% of people in Chile older than 15 were obese (1), and in 2019, 25% of the first-grade children in the country were obese (2). This trend coincided with the rising consumption of ultraprocessed foods and beverages (UPFs) (3), which in 2010 represented 28.6% of Chileans' total energy intake (4). Unhealthy food consumption patterns and nutritional statuses are the leading causes of deaths and disabilities in the country (5).

To address this problem the Chilean government implemented the Food Labeling and Advertising Law (hereinafter “the Law”). Since June 2016 foods and beverages with ingredients adding nutrients of concern (i.e., sugars, saturated fats, and sodium) that exceed established limits must display up to four front-of-package warning labels indicating that they are high in calories, total sugars, saturated fats, or sodium. The Law also prohibits selling regulated products in schools and advertising them to children under 14 years old. The Law was implemented in a staggered three-phase approach with the cutoffs for nutrients of concern becoming increasingly stricter in 2018 and 2019 (6).

To adapt to the regulations the food industry reformulated products and reduced the amounts of nutrients of concern, especially sugars (7, 8). For instance, after the first phase of implementation from 2016 to 2017, the prevalence of beverages high in sugars decreased from 26 to 11% (7). It is possible that the industry achieved this by replacing sugars partially or totally with non-nutritive sweeteners (NNSs).

A study conducted in 2019, after the third phase of the implementation, described NNS use among foods and beverages in Chile as frequent. More than half of that study's sample, composed of dairy products, cereal products (packaged bread, breakfast cereals, cereal bars, cookies, and packaged pastries), processed fruits, beverages, and sweets and desserts, presented an NNS (9). We hypothesized that this NNS use is related to the food law. However, only one study with very small sample of beverages (*n* = 7) suggested an increase in NNS use after the Law's implementation (10). as far as we know, no other study has examined changes in NNS use in the Chilean food and beverage supply.

This topic is important because although food reformulation can reduce consumption of nutrients of concern and prevent associated diseases (11), it also raises questions regarding the replacement ingredients and products' healthfulness (12). A food or beverage with an added NNS, for instance, is defined as a UPF (13), and UPF consumption has been associated with weight gain, obesity, and non-communicable diseases (14–16). A randomized controlled trial showed that a diet of UPFs resulted in greater energy intake and weight gain compared with a diet of minimally processed foods even though both diets were matched for macronutrients, energy, sugars, and fiber (17). In addition, we lack evidence that consuming NNSs in place of sugars is effective to achieve weight or metabolic control (18, 19), and it is not established whether long-term consumption of NNSs is safe, especially for children (20).

Considering that front-of-package regulations are becoming increasingly common as a strategy to reduce the consumption of foods and beverages with high amount of nutrients of concern, and the Chilean's model has been used to discuss and implement similar policies in other countries as Israel, Canada, Brazil, Peru, Uruguay, and Mexico (21), it is relevant to understand how this policy may affect the use of NNS in the food supply. This study aimed to evaluate changes in NNS use in Chile 7–8 months after the initial implementation of the Law. Specifically, we looked for changes in the prevalence of products with NNSs, in the number of different NNSs used, and in the frequency with which products added NNSs and sugars after the Law's implementation compared to the pre-implementation period. Additionally, we evaluated whether these changes were related to product reformulation to avoid a warning label.

## Materials and Methods

### Chilean Food and Beverage Database

We collected our data during January and February of 2015, 2016, and 2017 at six major supermarket chains (one store per chain) and three candy distributors in Santiago, Chile. The Chilean Supermarket Association granted permission to conduct the study. We photographed all sides of the packaged foods and beverages available from the stores and distributors, and trained dietitians recorded each product's general identifying information (i.e., barcode, brand, description, package size and type, manufacturer, etc.), the nutrition facts panel, and the list of ingredients in an online platform. Chile requires that packaged foods and beverages declare their nutrients and ingredients, including type and amount of each NNS, per serving size and per 100 grams (g) or 100 milliliters (ml) (22).

We pooled the data from 2015 and 2016 to construct a pre-implementation sample because a previous study had confirmed that food and beverage industries had made no anticipatory reformulations (22). The data collected in 2017 comprised the post-implementation sample. We excluded items without relevant information, such as a list of ingredients and a nutrition facts panel; products with a market share <1% of their food groups; and products recurring in multiple package sizes. We included only products with ingredients adding sugars, sodium, or saturated fats (i.e., products with added nutrients of concern and therefore subject to regulation) or products without any added nutrients of concern but with an NNS. From the total products collected in the three waves (*n* = 26,748), 4,715 were duplicated, 692 lacked relevant information, 4,071 were not under the scope of the regulation, and 11,564 were not best-selling products. The longitudinal sample was composed of all products for what matching was possible based on the barcode, brand, manufacturer name, and description of the products (*n* = 1,681). We classified each food and beverage into 1 of 16 mutually exclusive groups based on a previously used classification (7). Supplementary Table S1 presents examples of products in each group and the number of products in each group with an NNS in the pre-implementation and post-implementation periods. The analytic sample included only products collected in both periods in the seven food groups in which more than one product had an NNS: beverages, dairy-based beverages, yogurts, breakfast cereals, desserts and ice creams, candies and sweet confectioneries, and sweet spreads (*n* = 999). More details on data collection and food groups are available elsewhere (7, 22).

### NNS Types

We searched products' lists of ingredients for the eight Chilean approved NNSs, acesulfame-K, aspartame, cyclamate, saccharin, sucralose, neotame, alitame, and stevia (23). We created binary variables for the presence or absence of each NNS in each product in the database in both the pre- and the post-implementation periods. We considered the number of different types of NNSs in the same product an integer variable that could assume values from zero to eight, the number of Chilean approved NNSs. For products with NNSs we also considered a three-level categorical variable, one NNS, two NNSs, and three or more NNSs. We identified products that did not have NNSs in the pre-implementation period but did in the post-implementation period.

### NNSs Combined With Ingredients Adding Sugars

We searched products' lists of ingredients to identify those with ingredients adding sugars as defined in the Chilean Ministry of Health's official guidelines and described in detail in Kanter et al. (22). We classified all products with NNSs into two groups, NNS only and NNS combined with ingredients adding sugars.

### High in Sugars Status

We considered the total sugars per 100.0 g or 100.0 ml of a product ready for consumption, following the instructions for reconstitution when necessary. We classified a product as high in sugars if it contained an ingredient adding sugars, and more than the initial implementation cutoff, 22.5 g of total sugars per 100.0 g of solid food or 6.0 g per 100.0 ml of liquid foods and beverages.

### Data Analysis

We described categorical variables as frequencies. Given the longitudinal nature of the sample, we used the McNemar test to compare the frequency of products with NNSs in the pre- and post-implementation periods. To evaluate changes in the number of different NNSs used and in the combined use of NNSs and ingredients adding sugars, we considered only the products with at least one NNS (*n* = 377) in both periods and applied the Wilcoxon signed-rank test and the McNemar test, respectively.

We used a negative binomial regression to evaluate the association between avoiding the high in sugars status (i.e., a product with total sugars above the cutoff defined for high sugars in the pre-implementation and below this cutoff in the post-implementation period) and adding an NNS in the post-implementation period (i.e., a product had no NNSs in the pre-implementation period and had at least one in the post-implementation period).

We used Stata version 16.0 to perform all analyses, and we considered a *p*-value < 0.05 significant.

## Results

We found six of the eight Chilean approved NNS types in our food and beverage sample, sucralose, acesulfame-K, aspartame, stevia, saccharin, and cyclamate. In the pre-implementation period 37.9% of the sample had at least one NNS compared with 43.6% in the post-implementation period, a 15.0% increase (*p* < 0.001). From the pre-implementation period to the post-implementation period we observed significant increases in the use of at least one NNS in beverages (72.0 vs. 82.6%, *p* < 0.001), dairy-based beverages (50.0 vs. 66.7%, *p* = 0.008), yogurts (60.3 vs. 62.6%, *p* = 0.046), and desserts and ice creams (13.9 vs. 22.7%, *p* < 0.001) but no significant changes among breakfast cereals, candies and sweet confectioneries, and sweet spreads (Table 1).

**Table 1:** Changes in the frequency of NNS use between the pre- and post-implementation periods by food or beverage group.

**Food or beverage group**	**Pre (%)**	**Post (%)**	**Absolute change (%)**	**Relative change (% of pre)**	***P*-value**
All products (*n* = 999)	37.9	43.6	5.7	15.0	<0.001
Beverages (*n* = 236)	72.0	82.6	10.6	14.7	<0.001
Dairy-based beverages (*n* = 42)	50.0	66.7	16.7	33.3	0.008
Yogurts (*n* = 179)	60.3	62.6	2.3	3.7	0.046
Breakfast cereals (*n* = 54)	24.1	24.1	0.0	0.0	1.000
Desserts and ice creams (*n* = 216)	13.9	22.7	8.8	63.3	<0.001
Candies and sweet confectioneries (*n* = 204)	12.3	13.2	0.9	8.0	0.317
Sweet spreads (*n* = 68)	17.6	17.6	0.0	0.0	1.000

Sucralose was the most common NNS in Chilean products overall and in almost all categories. Acesulfame-K and aspartame followed sucralose in the overall ranking and were mostly used in beverages. We found stevia predominantly in dairy products and saccharin and cyclamate in only a small portion of products. The frequency of sucralose increased significantly by 26.2% relative to pre-implementation, and the frequency of stevia increased by 55.6%. Sucralose and stevia significantly increased in beverages and yogurts, whereas only sucralose increased in dairy-based beverages, desserts and ice creams, and sweet spreads (Table 2).

**Table 2:** Changes in the frequency of each type of NNS in the pre- and post-implementation periods by food or beverage group.

**Food or beverage group**	**Pre (%)**	**Post (%)**	**Absolute change (%)**	**Relative change (% of pre)**	***P*-value**
**Sucralose** (overall, *n* = 999)	24.8	31.3	6.5	26.2	<0.001
Beverages (*n* = 236)	43.2	54.7	11.5	26.5	<0.001
Dairy-based beverages (*n* = 42)	45.2	64.3	19.1	42.1	0.005
Yogurts (*n* = 179)	48.6	53.1	4.5	9.2	0.005
Breakfast cereals (*n* = 54)	13.0	11.1	−1.9	−14.3	0.317
Desserts and ice creams (*n* = 216)	7.4	15.3	7.9	106.2	<0.001
Candies and sweet confectioneries (*n* = 204)	4.4	5.4	1.0	22.2	0.317
Sweet spreads (*n* = 68)	11.8	17.6	5.8	50.0	0.046
**Acesulfame-K** (overall, *n* = 999)	18.8	19.4	0.6	3.2	0.180
Beverages (*n* = 236)	56.8	58.5	1.7	3.0	0.157
Dairy-based beverages (*n* = 42)	9.5	9.5	0.0	0.0	1.000
Yogurts (*n* = 179)	14.0	15.1	1.1	8.0	0.157
Breakfast cereals (*n* = 54)	1.9	0.0	−1.9	−100.0	0.317
Desserts and ice creams (*n* = 216)	3.7	5.1	1.4	37.5	0.083
Candies and sweet confectioneries (*n* = 204)	7.8	6.9	−0.9	−12.5	0.317
Sweet spreads (*n* = 68)	0.0	0.0	0.0	–	1.000
**Aspartame** (overall, *n* = 999)	14.4	14.1	−0.3	−2.1	0.257
Beverages (*n* = 236)	49.6	50.0	0.4	0.9	0.564
Dairy-based beverages (*n* = 42)	0.0	0.0	0.0	–	1.000
Yogurts (*n* = 179)	0.0	0.0	0.0	–	1.000
Breakfast cereals (*n* = 54)	0.0	0.0	0.0	–	1.000
Desserts and ice creams (*n* = 216)	3.7	3.7	0.0	0.0	1.000
Candies and sweet confectioneries (*n* = 204)	8.3	7.4	−0.9	−11.8	0.157
Sweet spreads (*n* = 68)	2.9	0.0	−2.9	−100.0	0.157
**Stevia** (overall, *n* = 999)	5.4	8.4	3.0	55.6	<0.001
Beverages (*n* = 236)	4.7	7.6	2.9	63.6	0.008
Dairy-based beverages (*n* = 42)	11.9	19.0	7.1	60.0	0.083
Yogurts (*n* = 179)	16.2	23.5	7.3	44.8	<0.001
Breakfast cereals (*n* = 54)	11.1	13.0	1.9	16.7	0.317
Desserts and ice creams (*n* = 216)	1.4	2.8	1.4	100.0	0.083
Candies and sweet confectioneries (*n* = 204)	0.0	0.0	0.0	–	1.000
Sweet spreads (*n* = 68)	0.0	4.4	4.4	–	0.083
**Saccharin** (overall, *n* = 999)	1.7	1.5	−0.2	−11.8	0.157
Beverages (*n* = 236)	0.8	0.8	0.0	0.0	1.000
Dairy-based beverages (*n* = 42)	0.0	0.0	0.0	–	1.000
Yogurts (*n* = 179)	0.0	0.0	0.0	–	1.000
Breakfast cereals (*n* = 54)	0.0	0.0	0.0	–	1.000
Desserts and ice creams (*n* = 216)	3.2	3.2	0.0	0.0	1.000
Candies and sweet confectioneries (*n* = 204)	2.9	2.9	0.0	0.0	1.000
Sweet spreads (*n* = 68)	2.9	0.0	−2.9	−100.0	0.157
**Cyclamate** (overall, *n* = 999)	1.4	1.3	−0.1	−7.1	0.317
Beverages (*n* = 236)	0.8	0.8	0.0	0.0	1.000
Dairy-based beverages (*n* = 42)	0.0	0.0	0.0	–	1.000
Yogurts (*n* = 179)	0.0	0.0	0.0	–	1.000
Breakfast cereals (*n* = 54)	0.0	0.0	0.0	–	1.000
Desserts and ice creams (*n* = 216)	2.8	2.8	0.0	0.0	1.000
Candies and sweet confectioneries (*n* = 204)	2.9	2.5	−0.4	−16.7	0.317
Sweet spreads (*n* = 68)	0.0	0.0	0.0	–	1.000

**Figure 1:**
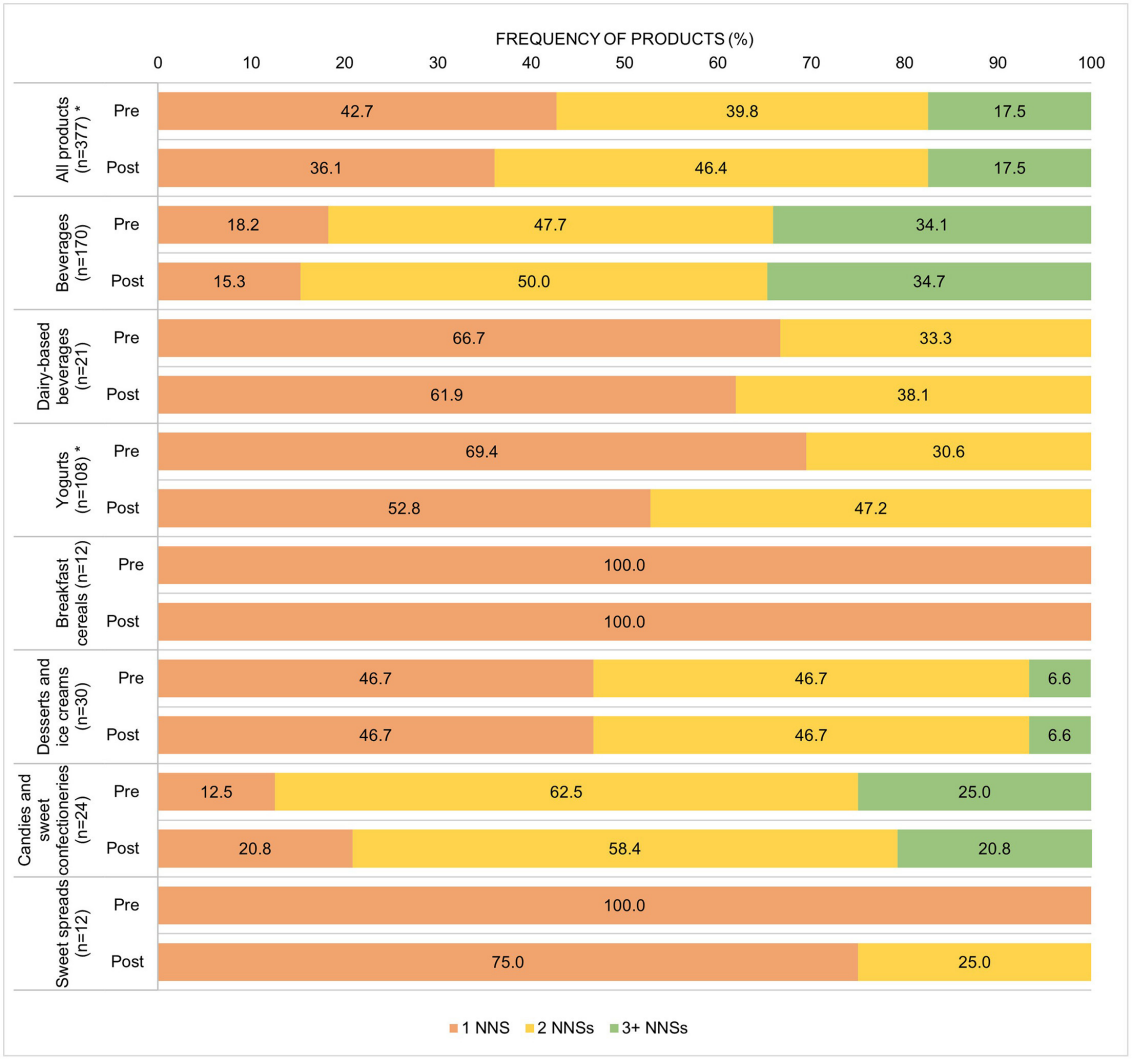
Number of different NNSs used in the pre- and post-implementation periods by food or beverage group. *p value < 0.001 in the Wilcoxon signed-rank test.

As Figure 1 shows, of the products that contained an NNS, 57.3% pre-implementation and 63.9% post-implementation had more than one type. We found up to four types of NNS in the same product. Only one beverage had four NNSs. Beverages and candies and sweet confectioneries especially used different types of NNSs. The number of NNSs increased in the post-implementation period in all products, and we found that a higher proportion of yogurts used two different NNSs.

**Figure 2:**
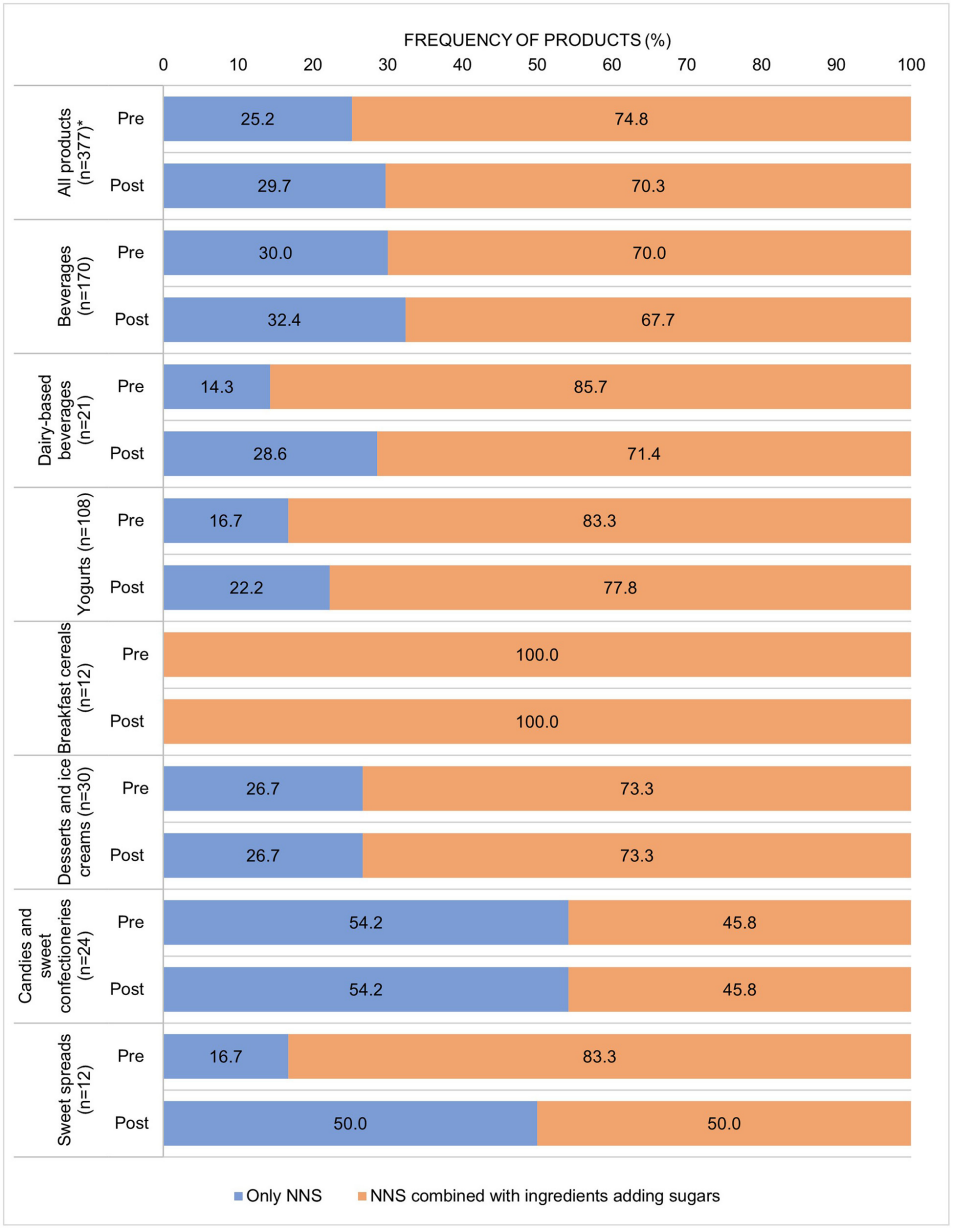
Use of NNSs only or NNSs in combination with ingredients adding sugars in the pre- and post-implementation periods by food or beverage group. *p value < 0.001 in the McNemar test.

Most products using any NNS (>70.0%) also included at least one ingredient adding sugars. The proportion of products with only an NNS and no ingredient adding sugars increased from 25.2 to 29.7%, but we did not identify significant changes among food or beverage groups (Figure 2).

In the pre-implementation period 39.3% of the products in the sample were classified as high in sugars given that they included one or more ingredients adding sugars and the amount of total sugars was greater than the Law's cutoff in the first phase of implementation. However, only 30.5% of the products were high in sugars after the implementation of the Law due to reformulation to decrease total sugars. We found that 34.4% of the reformulated products that changed from high in sugars to not high in sugars started using at least one NNS after the Law's implementation, whereas only 2.8% of the products that did not change their status on sugars started to use an NNS in the post-implementation period (prevalence ratio: 12.1; 95% confidence interval: 7.2–20.2; *p* < 0.001).

## Discussion

We examined a sample of foods and beverages offered in the main supermarket chains and candy distributors of Santiago, Chile, to determine for the first time changes in the use of NNSs after Chile implemented the Law. NNS use was high (above 1/3 of the studied sample) in Chile in 2015–2016 and increased 15% after the Law's initial implementation phase, particularly sucralose and stevia. We found significant increases in the proportion of NNSs in beverages, dairy-based beverages, yogurts, and desserts and ice creams with NNSs, despite the already high prevalence in those groups in the pre-implementation period. Foods and beverages that shifted away from a high in sugar status were 12 times more likely to initiate NNS use in the post-implementation period.

Compared with other countries, Chile has an elevated prevalence of products containing NNSs. We included in this study only the categories of sweet items in which 37.9% of the products used NNSs in the pre-implementation period. Among all of the products subject to regulation described in Supplementary Table S1, the frequency was 22.5% or 379 of 1,681 products with an NNS before implementation. This prevalence is more than twice that observed in Mexico in 2015–16 (11.0%) and much higher than those observed in the United States in 2015–17 (4.0%), New Zealand in 2016 (1.0%), and Australia in 2015 (1.0%) (24). The frequency of NNSs was especially high in beverages in which more than 70.0% of items added NNSs in the pre-implementation period and 80.0% in the post-implementation period. This is in line with a previous study's finding that 75.0% of beverages in Chile in 2019 included an NNS (9). This frequency is also higher than those observed in beverages in Mexico in 2015–16 (36.0%), the United States in 2015–17 (22.0%), New Zealand in 2016 (8.0%), and Australia in 2015 (3.0%) (24).

A previous report showed a significant decrease in the frequency of products high in sugars among beverages, dairy-based beverages, breakfast cereals, sweet baked products, candies and sweet confectioneries, and savory spreads in the post-implementation period (7). Our results suggest that at least to some extent food industries responded to Chile's 2016 Law by replacing sugars with NNSs. The association we found between industries reducing the amount of sugars to below the Law's threshold and starting to use NNSs supports this hypothesis. Measures focused on sugars reformulation, without considering the potential replacement by NNSs, could increase the dietary exposure to NNSs. In 2020, Mexico approved its front-of-package warning labels that include a note informing the presence of NNSs. This measure is a breakthrough in labeling policy and can help people better identify NNSs in products.

The use of an NNS is a strategy for maintaining a sweet taste with reduced or free calories. However, we found that most products with NNSs also had ingredients adding sugars (75% pre-implementation and 70% post-implementation), suggesting that NNS consumption does not eliminate sugars consumption. Additional analyses are needed to evaluate how NNS products impact the overall consumption of sugars. The total sugars content in products with NNSs varies between countries, probably because of factors like taste preferences (24). For instance, compared with three other countries (Australia, New Zealand and United States), Mexico showed the highest prevalence of NNSs in the food and beverage and the highest amount of total sugars in products with NNSs (24).

Using NNSs extensively and including more than one NNS in the same product contribute to the intake of a mixture of them. The food industry blends NNSs to enhance palatability (25), and we found that Chilean products commonly mixed two or three. A recent study on the French food and beverage supply described a similar situation with clusters of NNSs in such products as free-sugar gums and artificially sweetened beverages (26). Using NNS blends can be advantageous since it avoids high consumption of an individual type of NNS and evades one NNS's acceptable daily intake (ADI). However, the possible synergistic effect of intakes of mixed additives is unknown (26).

We also observed the frequency of each type of NNS in foods and beverages in Chile. Sucralose was the most frequent NNS, followed by acesulfame-K and aspartame. Another study found that sucralose, aspartame, and acesulfame-K were also the most common NNSs used in Brazil (27). We also identified changes during the period and noted an increasing use of sucralose and stevia. Some characteristics of sucralose could explain its frequent use, for example, the absence of a bitter aftertaste and stability at a high temperature and a low pH (28). Another study in Chile demonstrated that sucralose is commonly combined with stevia and other NNSs (9). We found that the prevalence of products containing stevia was 57.4% higher post-implementation compared with pre-implementation. This marked increase could be related to consumer's interest in natural products, since this NNS is an extract of the stevia plant (25, 29). Other countries have observed this tendency also. For instance, in the United States the prevalence of household purchases of products with rebaudioside A, a steviol glycoside, rose from virtually zero to 25.0% between 2002 and 2018 (29).

The increased use of NNSs in products children frequently consume, that is, beverages, dairy-based beverages, yogurts, and desserts and ice creams, may impact NNS intake among this specific population. Desserts, dairy products, and beverages accounted for ~30% of the calorie intake in a sample of Chilean preschoolers in 2016 (30) therefore those products are likely important NNS sources among this age group. NNS consumption was prevalent among Chilean children before implementation of the Law (31) and conceivably has increased after implementation. From March to June 2016, just before the Law was implemented, 68% of low- to medium-income preschoolers in Santiago consumed at least one NNS on the day of a dietary recall (31). In another study in Santiago in 2019, 3 years after the Law's first phase of implementation, all children 6–12 years old reported consumption of products containing NNSs, mostly dairy products and beverages (32). Despite widespread availability and consumption, the effects of early exposure to NNSs are not understood well and potentially could be linked with cardiometabolic risk in children (33).

Because consumption of sweet products, such as sugary beverages, is high in Chile, it is reasonable to suppose a populational preference for sweeter tastes (34). Food reformulation with widespread use of NNS, additives with intense sweetening power could maintain or increase the predilection for sweet tastes. Hence other policies should promote diets based on minimally processed and in natura in the country.

This study has some limitations. First, we were unable to test the causal effect of the Law's implementation on the use of NNSs in products. We cannot discount a prior trend driven by other policies, for example, the increase in the tax on sugar-sweetened beverages in 2014 (35), or a growing concern about sugar consumption, which could also impact food reformulation. In Colombia the amount of total sugars in beverages decreased and the proportion of beverages sweetened with NNSs increased between 2016 and 2018 (from 33 to 64%) without mandatory policies (36). However, in Chile the amount of nutrients of concern, including total sugars, in foods and beverages did not change before the Law's implementation (22), and the prevalence of products high in nutrients of concern decreased only after implementation (7). This evidence suggests the policy played a role in food reformulation in Chile. Moreover, we did not consider the market share in estimating weight-sales data, thus we cannot anticipate how the reported changes impact the populational intake of NNSs in Chile. Nonetheless, to get meaningful results we included only foods and beverages with more than 1% of the sales in their specific categories. Finally, we used the data available on the packages with no additional laboratory analyses, and we did not consider the use of other types of sweeteners, such as polyols. However, we collected data prospectively and used a relatively large longitudinal data set to better assess changes in NNS use.

A few months after Chile implemented its food law the amount of nutrients of concern, mainly sodium and total sugars, in the food supply declined and sweetened beverage consumption declined. The Chilean regulation is innovative because it combines a series of strategies, including front-of-package warning labels and marketing and sales restrictions, and other countries consider it a model. However, little is known about the substitutes that have replaced the nutrients of concern, particularly the NNSs as sugars substitutes. To our knowledge this study is the first to use prospectively collected data to examine changes in NNS use after implementation of the Chilean Law. Compared with the pre-implementation period, the post-implementation period saw an overall higher prevalence of products with NNSs.

Although the absolute differences we found were small, such as a 6% increase in NNS use, this study and previous reports suggest that at least in part NNSs, mostly sucralose and stevia, have replaced sugars after the implementation of the Law. We found that Chile's NNS use pre-implementation was high, which could preclude a larger increase in NNS frequency. Further studies should assess what happened after the final implementation of the Law and evaluate the possible consequences of similar measures in countries where the prior use of NNS is not as widespread. Some countries are including NNS restrictions in their policy discussions and designs. Following the Pan American Health Organization Nutrient Profile Model recommendations (37), Mexico recently implemented front-of-package warning labels that include a clear message regarding the presence of NNSs. Studies comparing the effects of these measures on local food reformulation are needed to guide future food policies.

## Conclusion

We identified increased NNS use in some food categories after the implementation of Chile's food labeling law in 2016. NNS use was frequent in the pre-implementation period, but in the post-implementation period NNS use significantly increased. It is necessary to further monitor NNS use as the Law's implementation applied stricter cutoffs in succeeding phases. Studies assessing the population's NNS intake and its impact are also needed to understand whether common NNS use should be a concern. Taken altogether, this evidence may suggest the need for food policies that regulate NNS use.

## Data Availability Statement

The datasets presented in this article are not readily available because the 2015-2017 nutritional information dataset was obtained upon a legal agreement made with the supermarket association ASACH. Such agreement includes a clause of not making publicly available the data. The Euromonitor International Database set is a commercial database that can be obtained upon payment from https://www.euromonitor.com/.

## Author Contributions

CZ: conceptualization, methodology, formal analysis, writing—original draft, and writing—review and editing. CC: conceptualization, writing—review and editing, and funding acquisition. LS: conceptualization and writing—review and editing. VQ: writing—review and editing. MR: conceptualization, methodology, supervision, and funding acquisition. All authors contributed to the article and approved the submitted version.

## Funding

This work was supported by the National Agency for Research and Development (ANID)/Scholarship Program/DOCTORADO BECAS CHILE (#2020 – 21200883); Bloomberg Philanthropies, International Development Research Center (IDRC) (#107731-002), and the ANID/Ministry of Heath Fondo Nacional de Investigación y Desarrollo en Salud (FONIS) (#SA19I0128). The funders had no role in study design, data collection and analysis, decision to publish, or preparation of the manuscript.

## Conflict of Interest

The authors declare that the research was conducted in the absence of any commercial or financial relationships that could be construed as a potential conflict of interest.

## Publisher's Note

All claims expressed in this article are solely those of the authors and do not necessarily represent those of their affiliated organizations, or those of the publisher, the editors and the reviewers. Any product that may be evaluated in this article, or claim that may be made by its manufacturer, is not guaranteed or endorsed by the publisher.
