# Supplementary material for: Changes in the Use of Non-nutritive Sweeteners in the Chilean Food and Beverage Supply After the Implementation of the Food Labeling and Advertising Law
Source: Front Nutr. 2021 Nov 8;8:773450. doi: 10.3389/fnut.2021.773450 (PMC8630583; doi:10.3389/fnut.2021.773450)
Supplement: Supplementary file 1 [file Table_1.DOCX]

Supplementary Material

Table S1. Descriptions of the 16 food and beverage groups and the number of items with an NNS.

| Food or beverage group (n) | Examples of specific foods or beverages | NNSs preimplementation (n) | NNSs postimplementation (n) |
| --- | --- | --- | --- |
| Beverages (n = 236) | Almond drinks with or without sugar, aromatized/flavored waters with or without sugar, carbonated waters with or without sugar, soft drinks with or without sugar, frozen and nonfrozen fruit pulps, fruit-based beverages (nectars and others) with or without sugar, coffee drinks with or without sugar, isotonic beverages (liquid and powder) with or without sugar, liquid teas with or without sugar, noncarbonated waters, powder/concentrated drinks with or without sugar, soy drinks with or without sugar | 170 | 195 |
| Dairy-based beverages (n = 42) | Liquid flavored semi-skimmed milk, liquid flavored skimmed milk, milk drinks (i.e., milk is beverages with at least 30% of the volume, according to the Chilean food regulations), powder semiskimmed milk, powder skimmed milk, powder whole milk, toddler milk (for children 1–5 years old) | 21 | 28 |
| Yogurts (n = 179) | Light or diet flavored yogurt, light or diet yogurt with fruits and/or nuts, light or diet yogurt with cereals, yogurt with fruits and/or nuts, yogurt with sweet sauces, yogurt with cereals, flavored yogurt, plain yogurt | 108 | 112 |
| Breakfast cereals (n = 54) | Baked corn cereals, chocolate cereals, chocolate cereal bars, dried fruit cereal bars, fiber-enriched cereals, flake/ball cereals, fruit cereal bars, granola, muesli, oatmeal, regular cereal bars | 13 | 13 |
| Sweet baked products (n = 108) | *Alfajores*, cookies, biscuits with filling, brownies, cakes, Chilean pastries, Christmas bread, *cuchuflies*, wafers with filling, frozen and nonfrozen *bizcochos*, muffins, sweet biscuits, wafers without filling, rolls | 0 | 1 |
| Desserts and ice creams (n = 216) | Canned fruits with or without sugar, cold desserts, diet ice creams, flans, frozen desserts, fruit and vegetable chips, fruit compotes, ice cream popsicles, roasted milks, milk rice puddings, water-based ice creams, water-based ice cream popsicles, yogurt ice creams | 30 | 49 |
| Candies and sweet confectioneries (n = 204) | Bonbons, bubble gum–filled candies, bubble gums, candy pops, chewy candies, chocolate-covered dried fruits, chocolates, caramel-covered dried fruits, gummies, jellies, liquid-filled bubble gums, caramel bars, caramel pops, marshmallows, sweet popcorns, toffees | 25 | 27 |
| Sweet spreads (n = 68) | Caramel spread, Chantilly cream, pastry creams, coconut cream, fresh cream, chocolate chips, chocolate sparks for pastry, *chuchoca*, condensed milk, evaporated milk, flavors (lemon, vanilla, etc.), flavored powders for milk, flavored syrups, frostings, grated coconut for pastry, honey, jam, sauces, pastry products | 12 | 12 |
| Savory baked products (n = 60) | Dough, frozen bread/dough, light or diet packaged white bread loaf, packaged white bread loaf, packaged whole wheat bread loaf, savory cookies, soda crackers, sopaipillas, wheat and corn tortillas | 0 | 1 |
| Savory snacks (n = 28) | Potato chips, *ramitas*, salty *soufflitos* | 0 | 0 |
| Savory spreads (n = 102) | Butter (regular and light), salad dressings, other dressings, chicken/meat/seafood broth, vegetable broth, chili pepper, ketchup, margarine (regular and light), mayonnaise, mustard, peanut butter, tomato sauce | 0 | 0 |
| Cheeses (n = 57) | Blue cheese, buttery cheese, cream, spreadable cheese, fresh cheese, grated cheese, light cheese, semihard cheese | 0 | 0 |
| Ready-to-eat meals (n = 83) | Canned vegetables, canned legumes, empanadas, frozen meals, *humitas*, instant mashed potatoes, meat substitutes, pasta-based preparations (e.g., macaroni and cheese), pizzas, potato gnocchi, ready-to-serve legumes, ready-to-serve vegetables, baby foods | 0 | 0 |
| Sausages (n = 116) | Bacon, chorizo, *choricillo*, ham, *longaniza*, salami | 0 | 0 |
| Nonsausage meat products (n = 71) | Fresh or frozen breaded meats; fresh or frozen breaded fish; canned seafood and fish; fresh or frozen marinated fish, chicken, pork, beef, or turkey; fish, chicken, pork, beef, or turkey hamburgers; fish, chicken, pork, beef, or turkey nuggets; other processed meats | 0 | 0 |
| Soups (n = 57) | Instant soups, legume and cream soups, powdered soups | 0 | 0 |
| **Total (n = 1,681)** | **All products** | **379** | **438** |

Adapted from Reyes et al. 2020.(7)
